# Pre-treatment clinical assessment in head and neck cancer: United Kingdom National Multidisciplinary Guidelines

**DOI:** 10.1017/S0022215116000372

**Published:** 2016-05

**Authors:** A Robson, J Sturman, P Williamson, P Conboy, S Penney, H Wood

**Affiliations:** 1 North Cumbria University Hospitals NHS Trust, Cumberland infirmary, Carlisle, UK; 2 Department of Anaesthesia, Cumberland Infirmary, Carlisle, UK; 3 Department of ENT Surgery, St George's Hospital NHS Trust, London, UK; 4 Department of Otolaryngology-Head and Neck Surgery, University Hospitals of Leicester, Leicester Royal Infirmary, Leicester, UK; 5 Department of Otolaryngology-Head and Neck Surgery, Manchester Royal Infirmary, Manchester, UK; 6 Department of Anaesthesia, Freeman Hospital, Newcastle upon Tyne NHS Foundation Trust, Newcastle upon Tyne, UK

## Abstract

This is the official guideline endorsed by the specialty associations involved in the care of head and neck cancer patients in the UK. This paper provides recommendations on the pre-treatment clinical assessment of patients presenting with head and neck cancer.

**Recommendations:**

• Comorbidity data should be collected as it is important in the analysis of survival, quality of life and functional outcomes after treatment as well as for comparing results of different treatment regimens and different centres. (R)

• Patients with hypertension of over 180/110 or associated target organ damage, should have antihypertensive medication started pre-operatively as per British Hypertension Society guidelines. (R)

• Rapidly correcting pre-operative hypertension with beta blockade appears to cause higher mortality due to stroke and hypotension and should not be used. (R)

• Patients with poorly controlled or unstable ischaemic heart disease should be referred for cardiology assessment pre-operatively. (G)

• Patients within one year of drug eluting stents should be discussed with the cardiologist who was responsible for their percutaneous coronary intervention pre-operatively with regard to cessation of antiplatelet medication due to risk of stent thrombosis. (G)

• Patients with multiple recent stents should be managed in a centre with access to interventional cardiology. (G)

• Surgery after myocardial infarction should be delayed if possible to reduce mortality risk. (R)

• Patients with critical aortic stenosis (AS) should be considered for pre-operative intervention. (G)

• Clopidogrel should be discontinued 7 days pre-operatively; warfarin should be discontinued 5 days pre-operatively. (R)

• Patients with thromboembolic disease or artificial heart valves require heparin therapy to bridge peri-operative warfarin cessation, this should start 2 days after last warfarin dose. (R)

• Cardiac drugs other than angotensin-converting enzyme inhibitors and angiotensin II antagonists should be continued including on the day of surgery. (R)

• Angotensin-converting enzyme inhibitors and angiotensin II antagonists should be withheld on the day of surgery unless they are for the treatment of heart failure. (R)

• Post-operative care in a critical care area should be considered for patients with heart failure or significant diastolic dysfunction. (R)

• Patients with respiratory disease should have their peri-operative respiratory failure risk assessed and critical care booked accordingly. (G)

• Patients with severe lung disease should be assessed for right heart disease pre-operatively. (G)

• Patients with pulmonary hypertension and right heart failure will be at extraordinarily high risk and should have the need for surgery re-evaluated. (G)

• Perioperative glucose readings should be kept within 4–12 mmol/l. (R)

• Patients with a high HbA1C facing urgent surgery should have their diabetes management assessed by a diabetes specialist. (G)

• Insulin-dependent diabetic patients must not omit insulin for more than one missed meal and will therefore require an insulin replacement regime. (R)

• Patients taking more than 5 mg of prednisolone daily should have steroid replacement in the peri-operative period. (R)

• Consider proton pump therapy for patients taking steroids in the peri-operative phase if they fit higher risk criteria. (R)

• Surgery within three months of stroke carries high risk of further stroke and should be delayed if possible. (R)

• Patients with rheumatoid arthritis should have flexion/extension views assessed by a senior radiologist pre-operatively. (R)

• Patients at risk of post-operative cognitive dysfunction and delirium should be highlighted at pre-operative assessment. (G)

• Patients with Parkinson's disease (PD) must have enteral access so drugs can be given intra-operatively. Liaison with a specialist in PD is essential. (R)

• Intravenous iron should be considered for anaemia in the urgent head and neck cancer patient. (G)

• Preoperative blood transfusion should be avoided where possible. (R)

• Where pre-operative transfusion is essential it should be completed 24–48 hours pre-operatively. (R)

• An accurate alcohol intake assessment should be completed for all patients. (G)

• Patients considered to have a high level of alcohol dependency should be considered for active in-patient withdrawal at least 48 hours pre-operatively in liaison with relevant specialists. (R)

• Parenteral B vitamins should be given routinely on admission to alcohol-dependent patients. (R)

• Smoking cessation, commenced preferably six weeks before surgery, decreases the incidence of post-operative complications. (R)

• Antibiotics are necessary for clean-contaminated head and neck surgery, but unnecessary for clean surgery. (R)

• Antibiotics should be administered up to 60 minutes before skin incision, as close to the time of incision as possible. (R)

• Antibiotic regimes longer than 24 hours have no additional benefit in clean-contaminated head and neck surgery. (R)

• Repeat intra-operative antibiotic dosing should be considered for longer surgeries or where there is major blood loss. (R)

• Local antibiotic policies should be developed and adhered to due to local resistance patterns. (G)

• Individual assessment for venous thromboembolism (VTE) risk and bleeding risk should occur on admission and be reassessed throughout the patients' stay. (G)

• Mechanical prophylaxis for VTE is recommended for all patients with one or more risk factors for VTE. (R)

• Patients with additional risk factors of VTE and low bleeding risk should have low molecular weight heparin at prophylactic dose or unfractionated heparin if they have severe renal impairment. (R)

## Introduction

This section deals with the topics of patient assessment and optimisation prior to treatment for head and neck cancer (HNC). The importance of collaborative teamwork, structured pre-operative assessment, grading and analysing comorbidity, and prophylaxis against infection and venous thromboembolism (VTE) are summarised in the section below.

## Comorbidity: outcomes and data collection

The presence of illnesses unrelated to the tumour significantly affects prognosis in HNC patients, and is contributed to by tobacco, alcohol and substance misuse. The Adult Comorbidity Evaluation 27 (ACE 27) and the Charlson Index are the most commonly used indices to quantify comorbidity.

The National Cancer Intelligence Network (NCIN) recommends that collection of an ACE 27 comorbidity score be mandated for all adult cancer patients. This facilitates surgical oncology research with the objective of improving cancer care through improved patient counselling and treatment planning. Information should be extracted from notes rather than relying on self-reporting.

Comorbidity scoring captures the impact of co-existing diseases, but not the disease of interest.^1^^,^^2^ Performance status assesses the effect of all illnesses on the patients' functional ability. Performance status is not a reliable substitute for comorbidity status as a prognostic measure, as they can each independently lead to poor tolerance of treatment. There is good evidence that integrating comorbidity with staging systems produces better prognostic instruments. The development of ‘prognostigrams’ relating to tumour–node–metastasis (TNM) stage, comorbidity and performance status require the accurate collection of these variables in large numbers as suggested by NCIN.

The effects of increased pre-treatment comorbid burden include:

• Adverse impact on short-term mortality of patients with newly diagnosed head and neck squamous cell carcinoma (HNSCC)
• Reduced overall survival in HNSCC and possible predictor for distant metastases
• Adverse influence on disease-specific survival, probably due to the advanced stage at presentation and the likelihood of such patients undergoing less aggressive treatment i.e. treatment selection
• Higher incidence of and more severe complications
• Adverse impact on quality of life (QoL)
• Adverse impact on functional outcomes
• Increased cost of treatment.

The relationship between performance status and survival is much less well-defined.

**Recommendation**

• Comorbidity data should be collected as it is important in the analysis of survival, QoL and functional outcomes after treatment as well as for comparing results of different treatment regimens and different centres. (R)

## Pre-operative assessment

A good pre-operative assessment system will provide an appropriately informed, consented and prepared patient on the day of surgery, avoiding late cancellation and preventable risk. It is imperative that referral for pre-operative assessment takes place as early as possible within the patient pathway.

Measures of the effectiveness of a pre-operative assessment service should be regularly audited. These include:

• Avoiding delay in listing and admission for surgery
• Avoiding unnecessary or duplicate investigations
• High proportion of same day admissions for surgery
• No cancellations as a result of inadequate investigation or workup
• Length of hospital stay.

The role of the anaesthetist in pre-operative assessment includes:

• Identification of the difficult airway
• Risk stratification and discussion
• Optimisation of comorbidities within the limited timeframe prior to surgery
• Formulation of a plan for peri-operative care with appropriate allocation to critical care resources.

Guidance for the use of pre-operative testing is available from National Institute for Health and Care Excellence (NICE), The Clinical Audit and Practice Advisory Group of ENT UK and the British Association of Day Surgery and Royal College of Anaesthetists.^3^^,^^4^ Individual department guidelines should be developed, including the use of general and dynamic testing.

There should be a clinical lead in each anaesthetic department for pre-operative assessment and for head and neck anaesthesia with established links to related specialities.

### Identification of the difficult airway

In head and neck practice, the surgeon and anaesthetist have an important role in identifying the difficult airway (Box I).

BOX I **RISK FACTORS FOR A DIFFICULT INTUBATION INCLUDE**

• Previous difficult intubation
• Mallampati grade III or IV
• Thyromental distance <6 cm
• Reduced mouth opening, inter-dental distance <3 cm
• Reduced neck extension
• Presence of retrognathia
• Poor dentition
• Obstructive laryngeal tumours
• Tongue base tumours
• Hypopharyngeal lesions
• Previous head and neck surgery or radiotherapy

A collaborative approach and communication greatly reduces the risk associated with a difficult airway. Suspected cases should be discussed between surgeon and anaesthetist prior to the day of surgery ideally with nasolaryngoscopy and scans to aid decision making. Airway assessment is imperfect in predicting problems and an airway strategy that encompasses emergency options should be formulated for both induction and the end of surgery. This strategy must be communicated clearly to the entire team working in theatre on the day and human factors considered.

### Risk stratification and optimisation of comorbidities

#### Assessment of risk

In recent years, there has been an increasing focus on risk prediction in patients undergoing major surgical procedures. In terms of risk stratification, head and neck surgery is classed as intermediate-risk surgery.

The POSSUM (Physiological and Operative Severity Score for the Enumeration of Mortality and Morbidity) score is a useful aid to predicting morbidity but despite being a well-validated tool, it has not demonstrated effective prediction of mortality in head and neck surgery.

The extensively validated Revised (Lee) Cardiac Risk Index (Box II) is a six point index score derived from patients over the age of 49; it is used to assess the risk of major cardiac event associated with non-cardiac surgery. This and other scoring systems were predominantly validated in the general and vascular surgical populations, but evidence suggests that it is a useful predictor of cardiovascular morbidity peri-operatively in head and neck surgery, particularly when combined with age over 70 as an additional variable.

BOX II **REVISED (LEE) CARDIAC RISK INDEX VARIABLES**

• History of IHD
• History of congestive heart failure
• Cerebrovascular disease (stroke or transient ischaemic attack)
• Diabetes requiring insulin use
• Chronic kidney disease (Creatinine > 2.0 mg/dl or 177 µmol/l)
• High-risk surgery – intra-peritoneal, intra-thoracic and suprainguinal vascular

Predicted risk of major cardiac event:

0 variables = 0.4 per cent risk

1 variable = 0.9 per cent risk

2 variables = 6.6 per cent risk

3 or more variables = 11 per cent risk

The assessment of dynamic function or aerobic fitness is extremely important to aid quantification of risk and allocation of critical care resources. Simple subjective methods include the estimate of metabolic equivalents (METs), where one MET equates to the oxygen consumption of a 70 kg man at rest, four METs equate to walking up one flight of stairs; failure to achieve this is associated with increased risk. Dynamic testing of functional capacity may include the use of shuttle walk testing, 6 minutes walk testing, treadmill cardiac testing and cardiopulmonary exercise testing (CPET).

CPET is currently the most reliable and objective assessment of functional capacity; the anaerobic threshold and peak oxygen consumption are proven to be well correlated with morbidity and mortality in the peri-operative period in major surgery; its application to head and neck surgery is still being evaluated.

The following sections will concentrate on identification of significant comorbidities and therapies which require specific pre-operative management, using a system-based approach.

#### Cardiovascular system

Between 40 and 50 per cent of patients will have cardiovascular disease.^5^ All patients over 55 years and those with diabetes or other cardiac risk should have an electrocardiogram (ECG) as a minimum pre-operative investigation.

##### Hypertension

Hypertension is the commonest cardiovascular comorbidity. There is evidence that hypertension with target organ damage is associated with a small increased incidence of major cardiovascular events.

The diagnosis of hypertension should be made in primary care. A patient with a blood pressure of greater than 180/110 mm Hg has severe hypertension and should not proceed to non-urgent surgery until the blood pressure is controlled to below 160/100. Patients with more moderate hypertension with associated target organ damage are also at higher risk. Where surgery must proceed patients should be made aware of the increased risk. Patients with hypertension demonstrate a more labile haemodynamic response to induction, airway instrumentation, surgical stimulus and post-operative pain. The practice of rapidly correcting pre-operative hypertension with beta blockade appears to cause higher mortality due to stroke and hypotension as per the PeriOperative ISchaemic Evaluation (POISE) study.

Patients with hypertension pre-operatively should be managed by their primary care doctor with introduction of antihypertensive agents as per the British Hypertension Society (BHS) guidelines.^6^

##### Ischaemic heart disease

Patients with poorly controlled ischaemic heart disease (IHD) should be referred for cardiology assessment. There is no evidence that pre-operative percutaneous coronary intervention (PCI) improves outcome, peri-operative nor long term, in patients with stable coronary artery disease; however, it is justifiable when it is likely to improve the patient's long-term prognosis, such as due to the presence of left main stem stenosis, three-vessel disease or left ventricular (LV) dysfunction. Referral to a cardiologist for patients with recent unstable coronary symptoms should precede surgery. If PCI is performed prior to major surgery bare metal stents are preferred, as these require only four to six weeks of dual antiplatelet therapy, which otherwise markedly increases peri-operative bleeding. Patients with poorly controlled IHD (including recent myocardial infarction (MI)) or who have had recent intervention should undergo surgery in a centre with access to interventional cardiology.

##### Cardiac investigations

Echocardiography is indicated in the situations shown in Box III.^7^

BOX III **INDICATIONS FOR ECHOCARDIOGRAM**

• Documented IHD with reduced functional capacity unexplained by other musculoskeletal disease
• Dyspnoea without obvious non-cardiac cause (e.g. METs < 4 or METs < 7 and dyspnoea with normal PFTs)
• Murmur or history of murmur PLUS symptoms suggestive of valve disease or abnormal ECG
• Known significant valve disease with change of symptoms or no echo within two years
• New atrial fibrillation
• New left bundle branch block or left ventricular hypertrophy on ECG
• Suspected cardiomyopathy
• Lung disease with suspicion of cardiac involvement (cor pulmonale)
• Known or suspected pulmonary hypertension

Dobutamine stress echocardiography can provide a useful dynamic assessment if IHD is suspected and CPET is not possible. Patients with severe valvular disease have an increased risk of surgery. Aortic stenosis can progress rapidly in the elderly population, and those with critical aortic stenosis may need to be considered for pre-operative intervention.

##### Arrhythmias and pacing

Atrial fibrillation and other arrhythmias are frequently found at pre-operative assessment; these may be known or new. Management should focus on the rate control, appropriate anticoagulation and identification of associated risks such as structural heart disease or indication for pre-operative pacing.

Cardiac pacemakers should have a recent battery and threshold check within one year. Patients with implantable defibrillators require organisation with a cardiology technician so that they can be deactivated in the anaesthetic room and external pads placed; this is due to the risk of inappropriate discharge due to anaesthetic drugs (suxamethonium) or movement. In both cases, theatre alerts should be placed to remind staff about diathermy risk and bipolar used.

##### Cardiac and anticoagulant drugs

Clopidogrel should normally be discontinued 7 days pre-operatively; aspirin should be continued without interruption. Patients taking warfarin for uncomplicated atrial fibrillation can discontinue it 5 days pre-operatively, restarting post-operatively when enteral function returns and the risk of bleeding is low. Patients with thromboembolic disease or artificial heart valves require heparin therapy to bridge peri-operative warfarin cessation. This will normally be with therapeutic dose low molecular weight heparin (LMWH) and can be managed in the community either with self-injection or district nurse involvement. Last dose should be 24 hours before the start of surgery. Patients with severe renal impairment will require adjusted dosing or occasionally unfractionated heparin infusion. LMWH or heparin infusion will need to be continued post-operatively until the INR is within the therapeutic range.

Newer oral anticoagulants (e.g. Dabigatran and Apixaban) have variable elimination times depending on renal and liver function; these are non-reversible agents and if there are no locally agreed policies, advice should be sought from a haematologist.

There is increasing evidence that statin therapy should be continued without interruption to prevent peri-operative coronary syndromes due to its plaque stabilising properties.

Provision should be made for enteral administration of cardiac drugs as early as possible post-operatively, and patients should continue the majority of these medicines up to admission. Angotensin-converting enzyme (ACE) inhibitors and angiotensin II antagonists are a source of debate; the majority of anaesthetists will choose to omit these drugs on the morning of surgery, particularly if it is purely for hypertension control.

**Recommendations**

• Patients with hypertension of >180/110 or associated target organ damage, should have antihypertensive medication started pre-operatively as per BHS guidelines (R)
• Rapidly correcting pre-operative hypertension with beta blockade appears to cause higher mortality due to stroke and hypotension and should not be used (R)
• Patients with poorly controlled or unstable IHD should be referred for cardiology assessment pre-operatively (G)
• Patients within one year of drug eluting stents should be discussed with the cardiologist who was responsible for their PCI pre-operatively with regard to cessation of antiplatelet medication due to risk of stent thrombosis (G)
• Patients with multiple recent stents should be managed in a centre with access to interventional cardiology (G)
• Surgery after MI should be delayed if possible to reduce mortality risk (R)
• Patients with critical AS should be considered for pre-operative intervention (G)
• Clopidogrel should be discontinued 7 days pre-operatively; warfarin should be discontinued 5 days pre-operatively (R)
• Patients with thromboembolic disease or artificial heart valves require heparin therapy to bridge peri-operative warfarin cessation; this should start 2 days after last warfarin dose (R)
• Cardiac drugs other than ACE inhibitors and angiotensin II antagonists should be continued including on the day of surgery (R)
• Angotensin-converting enzyme inhibitors and angiotensin II antagonists should be withheld on the day of surgery unless they are for the treatment of heart failure (R)
• Post-operative care in a critical care area should be considered for patients with heart failure or significant diastolic dysfunction (R)

##### Heart failure and diastolic dysfunction

Heart failure is a considerably greater peri-operative risk factor than angina or previous MI alone. New or poorly controlled heart failure should be referred to cardiology for optimisation, with early commencement and uptitration of an ACE inhibitor, unless contraindicated, whilst that assessment is pending.

Heart failure carries a 50 per cent four-year mortality from diagnosis if the underlying cause cannot be treated; 50 per cent of patients with severe heart failure (symptomatic and frequent presentations) will die within one year.

Post-operative care in a critical care area should be considered for patients with heart failure or significant diastolic dysfunction.

Right heart failure carries a very high peri-operative risk, much more than LV failure, and there is little available treatment. Right heart failure associated with pulmonary hypertension carries extraordinary risk and is discussed in the respiratory section.

#### Respiratory system

Significant respiratory disease occurs in 20–30 per cent of patients and respiratory morbidity is the most frequent medical complication of major surgery and cause of intensive care unit stay. Preoperative respiratory disease should be optimised wherever possible and right heart disease and pulmonary hypertension considered in those with significant hypoxia (oxygen saturations <93 per cent) or exercise limitation.

##### Respiratory investigations

Chest radiographs are not required routinely from a fitness perspective; the functional capacity of the patient is paramount here. Cardiopulmonary exercise testing may be useful to assess dynamic function and can demonstrate whether respiratory disease is the main contributing factor to generalised debility.

Intensive care must be planned for patients with significant pulmonary hypertension.

Lung disease should be quantified with spirometry and in severe cases arterial blood gas sampling. An FEV1 of less than 25 per cent predicted poses a markedly increased risk of post-operative ventilatory support, especially when accompanied by hypoxia, hypercarbia or cor pulmonale. The risk of respiratory mortality alone may outweigh any benefit from major surgery. The following actions will optimise a patient's condition for surgery:

• Optimise bronchodilator therapy
• Trial of steroid responsiveness in moderate and severe disease
• Smoking cessation
• Peri-operative nebuliser therapy
• Treatment of inter-current chest infection, possibly delaying surgery
• Sputum sampling to enable ‘best guess’ treatment of chest infection.

Patients with significant hypoxia (oxygen saturations greater than 93 per cent) arterial blood gas estimation should be performed to look for CO_2_ retention. Such patients should be considered for an echocardiogram.

##### Obstructive sleep apnoea

It is useful to know the degree of obstructive sleep apnoea (OSA) pre-operatively to allow post-operative care planning and to consider the need to exclude pulmonary hypertension and right heart failure, which may occur if there has been an extended period of untreated OSA. Mask fitting should be optimised and any change to anatomy that could compromise use of the mask should be considered and managed appropriately. Patients with proven or suspected OSA and no continuous positive airway pressure (e.g. not tolerated), will require critical care for at least the first post-operative night.

**Recommendations**

• Patients with respiratory disease should have their peri-operative respiratory failure risk assessed and critical care booked accordingly (G)
• Patients with severe lung disease should be assessed for right heart disease pre-operatively (G)
• Patients with pulmonary hypertension and right heart failure will be at extraordinarily high risk and should have the need for surgery re-evaluated (G)

#### Endocrine system

##### Diabetes

Poor glycaemic control is associated with increased wound infections, post-operative morbidity, intensive care requirements and hospital mortality. Peri-operative glucose readings should be kept within the target range of 6–10 mmol/l or the acceptable range of 4–12 mmol/l in order to reduce risk. HbA1C is a useful indicator of diabetic control within the preceding three months and patients with an HbA1C greater than 69 should be considered as higher risk of peri-operative poor glucose control. If time allows, these patients should be referred to a diabetes specialist as important changes can be made to glucose control within two to three weeks of surgery.

Clear and accessible peri-operative diabetes guidelines should be available in every hospital; National Health Service (NHS) guidelines are available for the management of adults with diabetes undergoing surgery.^8^ Insulin-dependent diabetic patients must not omit insulin for more than one missed meal and will therefore require an insulin replacement regime, such as a variable rate intra-venous insulin infusion (VRIII) or a glucose potassium insulin infusion (GKI) for major surgery. Many of the longer acting insulins regimes should be continued at reduced dose alongside the insulin replacement regime. Oral hypoglycaemic agents should be omitted on the day of surgery and restarted when normal diet is resumed. Many of these patients will also require a VRIII or GKI. This can still be managed with day of surgery admission in the well-controlled diabetic patient, provided that sufficient protocols are in place.

Many patients with HNC will have an adjusted diet post-operatively and input from diabetic specialists is important to successfully manage medication requirements.

##### Steroids

Steroid replacement is essential for those with adrenal suppression from primary or secondary causes to prevent potentially fatal adrenal crises; 60 per cent of patients taking 5 mg of prednisolone daily fail a short Synacthen test and are therefore at risk of relative post-operative adrenal failure. Guidelines agreed and awaiting publication by the AAGBI and agreed by the Clinical Advisory Panel to the Addison's Disease Self-Help Group, recommend the use of peri-operative steroid cover for all patients taking more than 5 mg prednisolone daily, or the equivalent doses of hydrocortisone 20 mg or dexamethasone 1 mg. Patients using inhaled, intra-nasal or topical steroids may also be at risk. Hydrocortisone is only therapeutic for 2–3 hours after intra-venous bolus, so the more traditional QDS bolusing can leave patients sub-therapeutic for several hours before the next dose.

The recommended steroid replacement regime is as follows: 100 mg IM hydrocortisone at induction followed 4 hours later by 200 mg by intra-venous infusion over 24 hours; this may be commenced intra-operatively. This infusion should be continued until oral steroids can be used. Oral dosing should be doubled for at least 48 hours for major surgery and then rapidly tapered back to normal dosing. If intra-venous infusion is impossible, a secondary option is IM 50 mg hydrocortisone QDS.

National Institute for Health and Care Excellence guidelines regarding oral corticosteroids note a higher risk of gastrointestinal bleeding and dyspepsia if steroid use is associated with advanced cancer, older age, concomitant non-steroidal anti-inflammatory medications or anticoagulants, previous gastrointestinal ulcer, bleed or perforation. These patients should be considered for proton pump therapy.

**Recommendations**

• Peri-operative glucose readings should be kept within 4–12 mmol/l (R)
• Patients with a high HbA1C facing urgent surgery should have their diabetes management assessed by a diabetes specialist (G)
• Insulin-dependent diabetic patients must not omit insulin for more than one missed meal and will therefore require an insulin replacement regime (R)
• Patients taking more than 5 mg of prednisolone daily should have steroid replacement in the peri-operative period (R)
• Consider proton pump therapy for patients taking steroids in the peri-operative phase if they fit higher risk criteria (R)

#### Neurological system

##### Stroke

Peri-operative stroke occurs in approximately 1 in 1000 patients with no prior history of stroke. The comparative odds ratios increase markedly in the presence of prior stroke.^9^

Where surgery cannot be delayed, attention must be paid to cardiovascular stability with avoidance of significant hypotension and head positioning to avoid compression or distortion of the neck vessels, which may impede cerebral perfusion pressure. Carotid dopplers are appropriate for stroke within 12 months.

##### Rheumatoid arthritis related neck instability

Patients with rheumatoid arthritis are at risk of atlanto-axial subluxation and subsequent cord injury and extreme caution should be used at intubation and head positioning.^10^ There are no clear guidelines on the use of cervical spine radiographs pre-operatively. Symptoms suggesting a higher risk of atlanto-axial instability include hesitation on neck movement, pain on movement radiating to the occiput, paraesthesia to the shoulder blades on head movement, or sensory loss in the hands. Up to 20 per cent of patients with rheumatoid arthritis can demonstrate abnormalities on radiographs and in view of the movement often required at surgery for head and neck disease it is advisable that these patients should have cervical spine stability assessed radiologically. Flexion and extension views of the cervical spine are required and should be interpreted by a senior radiologist.

**Recommendations**

• Surgery within three months of stroke carries high risk of further stroke and should be delayed if possible (R)
• Patients with rheumatoid arthritis should have flexion/extension views assessed by a senior radiologist pre-operatively (R)
• Patients at risk of POCD and delirium should be highlighted at pre-operative assessment (G)
• Patients with Parkinson's disease (PD) must have enteral access so drugs can be given intra-operatively. Liaison with a specialist in PD is essential (R)

##### Post-operative cognitive dysfunction (POCD) and post-operative delirium

Post-operative cognitive dysfunction is new cognitive impairment arising after a surgical procedure, which may be permanent. The incidence of POCD in non cardiac surgery is in the region of 20 per cent at one week and 10 per cent at three months, rising with age. The incidence of delirium (temporary acute confusional state) is higher.

Every effort should be made to highlight these risk factors at pre-operative assessment so the anaesthetic and post-operative care can be tailored accordingly (Box IV). This may include the use of short acting anaesthetic agents, close monitoring for infection and ensuring adequate pain relief; but also includes ensuring the patient has all necessary aids such as for hearing and sight.

BOX IV **RISK FACTORS FOR POCD OR DELIRIUM INCLUDE**

• Age > 70
• Preoperative cognitive impairment or dementia
• Depression
• Preoperative alcohol misuse
• Visual impairment
• Renal dysfunction
• Tobacco use
• Previous delirium

#### Haematological system

The commonest haematological abnormality is anaemia, usually due to iron deficiency. It is essential that haematinic evaluation is completed to look for the specific deficiency, which may be associated with nutritional failure. A source of iron deficiency anaemia should be always sought (occult malignancy, ulcer disease).

Treatment should be based on the active replacement of the haematinics, whether B_12_, folate or iron. Iron replacement can be oral or intravenous; oral therapy will only be effective if absorption is likely and there is at least six weeks before surgery. Intravenous iron is increasingly used and can cause a meaningful rise in haemoglobin levels within two to three weeks. Erythropoietin should be considered on advice from a haematologist or nephrologist for patients with anaemia due to renal disease or anaemia related to chronic disease.

**Recommendations**

• Intravenous iron should be considered for anaemia in the urgent HNC patient (G)
• Preoperative blood transfusion should be avoided where possible (R)
• Where pre-operative transfusion is essential it should be completed 24–48 hours pre-operatively (R)

Preoperative transfusion should be avoided wherever possible and considered on a case by case basis rather than a target haemoglobin level. There is no evidence to support a cut off transfusion point and there is significant risk independently associated with peri-operative blood transfusion. Where pre-operative transfusion cannot be avoided, it should be completed at least 24–48 hours pre-operatively in order to allow time for regeneration of 2,3-diphosphoglycerate in stored red cells, which ensures optimum oxygen delivery by the haemoglobin.

### Alcohol and smoking

#### Alcohol misuse

There is an increased rate of high alcohol intake in patients with HNCs. General post-operative complication rates are approximately 50 per cent higher for patients who drink 5–6 units of alcohol per day compared with those who drink 0–3 units. If untreated, 6 per cent of alcohol-dependent patients will develop clinically relevant symptoms of withdrawal, and up to 10 per cent of these will experience delirium tremens. Acute alcohol withdrawal in the context of major surgery can cause significant morbidity and a peri-operative mortality of up to 10 per cent.

An accurate alcohol assessment should include details of intake and level of dependency as well as impact on general health. Alcohol withdrawal should be considered in any patient who has hazardous drinking levels defined as more than 5 units per day for men, 3 units per day for women. Information about appropriate alcohol counselling and support should be provided to patients considered at risk.

Identification of alcohol dependency at pre-operative assessment enables further investigation for associated conditions. It also allows planning for pre-operative detoxification, prophylactic intervention, and a higher level of vigilance during admission for the early signs of alcohol withdrawal.

Patients considered to have a high level of dependency should be considered for active in-patient withdrawal at least 48 hours pre-operatively in liaison with relevant specialists.

Thiamine deficiency is common in patients with alcohol dependency and oral absorption can be poor. Parenteral B vitamins (Pabrinex) should be used before surgery to prevent Wernicke–Korsakoff syndrome in those patients with high levels of alcohol intake.

#### Tobacco use

Smoking tobacco before diagnosis in patients with HNC has a negative correlation with survival. A significant proportion of patients attending cancer diagnostic clinics are smokers. Continued tobacco use in the period leading up to surgery is associated with higher morbidity and mortality in general. Smokers have a considerably increased risk of both intra-operative and post-operative complications, including a 3 to 6-fold increase of peri-operative pulmonary complications. Patients requiring flap reconstructions have higher flap failure rates and greater wound infection rates. Continued smoking during radiotherapy treatment increases complications in patients with laryngopharyngeal cancer and increases the risk of treatment failure. Smoking shortens overall survival and increases both the risk of recurrence and of developing a second primary tumour.

Ideally patients should be supported to stop smoking from the time of their initial clinic visit. Stopping for 24–48 hours pre-operatively normalises the amount of carbon monoxide in the blood, which may be as high as 15 per cent in smokers, allowing better oxygen carrying capacity of the blood to the heart and surgical wounds. Stopping for four to six weeks will allow the immune system recovery. Stopping for six to eight weeks allows recovery of respiratory tract cilia function. Nicotine withdrawal should be treated both pre- and post-operatively.

The UK Government has set up a comprehensive NHS Stop Smoking Service and a range of products and interventions are available. Many trusts now use the successful Stop Before Your Op campaign.

**Recommendations**

• An accurate alcohol intake assessment should be completed for all patients (G)
• Patients considered to have a high level of alcohol dependency should be considered for active in-patient withdrawal at least 48 hours pre-operatively in liaison with relevant specialists (R)
• Parenteral B vitamins should be given routinely on admission to alcohol-dependent patients (R)
• Smoking cessation, commenced preferably six weeks before surgery, decreases the incidence of post-operative complications (R)

### Nutritional failure

Nutritional failure impacts negatively on mortality, infection and wound healing. Detailed nutritional assessment and support should be instituted pre-operatively for all patients facing major head and neck surgery as a matter of routine. Dietician-led nutritional support intervention should be provided to any at-risk patients as part of their multidisciplinary management.

## Antibiotic prophylaxis

The rationale for considering surgical antibiotic prophylaxis is based on reducing major morbidity, reducing patient length of stay, reducing hospital costs and decreasing overall consumption of antibiotics. Antibiotic use is not without risk and careful adherence to local antibiotic policies is essential to account for local resistance patterns.

Risk factors affecting the incidence of surgical site infection can be both patient and operation associated. Head and neck cancer patients who smoke, are obese (over 20 per cent of ideal body weight), diabetic and immunosuppressed, and have advanced disease or require free flap reconstruction have the greatest risk of surgical wound infection. Operative factors include duration of surgery, antimicrobial prophylaxis and surgical technique (haemostasis, appropriate use of drains, tissue handling and wound closure). The risks of infection can be minimised by:

• Day of surgery admission where possible
• Advising patients to shower or bathe on the day before or the day of surgery
• Methycillin-resistant *Staphylococcus aureus* screening and use of topical agents to reduce carriage if required
• Use of antibiotic prophylaxis, where indicated
• Aseptic surgical technique and careful tissue handling
• Minimising post-operative stay.

The Scottish Intercollegiate Guidelines Network has published a review of the role of antibiotic prophylaxis in surgery, updated in April 2014. Whilst the guidelines are for surgery in general, the search criteria and conclusions include evidence and specific conclusions for head and neck surgery. It must be remembered that antibiotic use is not without risk and reducing inappropriate prescribing is one of the aims of rationalising surgical antibiotic prophylaxis.

In the setting of clean head and neck surgery for benign disease, antibiotic prophylaxis is not recommended. For surgery with malignant disease that is clean (e.g. neck dissection) antibiotic prophylaxis can be considered. For contaminated and clean-contaminated surgery antibiotic prophylaxis is recommended. In this setting, a single dose of antibiotic with a long enough half-life to achieve activity throughout the operation is recommended. The duration of prophylactic antibiotics should not be more than 24 hours. The choice of antibiotic should ensure broad-spectrum cover for aerobic and anaerobic organisms.

**Recommendations**

• Antibiotics are necessary for clean-contaminated head and neck surgery, but unnecessary for clean surgery (R)
• Antibiotics should be administered up to 60 minutes before skin incision, as close to the time of incision as possible (R)
• Antibiotic regimes longer than 24 hours have no additional benefit in clean-contaminated head and neck surgery (R)
• Repeat intra-operative antibiotic dosing should be considered for longer surgeries or where there is major blood loss (R)
• Local antibiotic policies should be developed and adhered to due to local resistance patterns (G)

The timing of the administration of prophylactic antibiotics is important. Intravenous antibiotic should be given up to 60 minutes before the skin is incised. There is some evidence which suggests this dose should be as close to incision as possible. Repeat dosing should be considered when the operation is significantly longer than the half-life of the antibiotic given. In the event of major intra-operative blood loss (>1500 ml), additional prophylactic antibiotic dosage should be considered after fluid replacement to maintain serum concentrations.

## Thromboembolic disease prophylaxis

The stated incidence of clinically significant venous thromboembolism (VTE) varies from 0 to 13 per cent in HNC operations.^11^ Variation may relate to extent of surgery, and non-pharmacological mechanical interventions, with most series showing an incidence of less than 1 per cent. Early mobilisation and adequate hydration status are essential therapeutic interventions.

Current NICE guidance on VTE and Scottish Intercollegiate Guidelines Network guidelines cover all surgical patients without specific reference to head and neck cases. Individual assessment for risk of VTE and bleeding should occur on admission and be repeated at least every 48 hours throughout admission.

All patients with one or more of the risk factors (Box V) should receive mechanical prophylaxis from admission (anti-embolism stockings to knee or thigh, or foot impulse devices or intermittent pneumatic compression devices) unless contraindicated. Do not offer anti-embolism stockings to patients with cardiac failure, peripheral arterial disease or neuropathy or local tissue damage. Patients should be encouraged to mobilise and remain well hydrated.

BOX V **RISK FACTORS FOR VENOUS THROMBOEMBOLISM INCLUDE**

Advancing age >60 years

Obesity – BMI > 30 kg/m^2^

Varicose veins

Family history of VTE

Thrombophilias

Presence of cancer or other thrombotic states

Significant medical comorbidities including heart disease

Oestrogen containing drugs including hormone replacement therapy or tamoxifen

Immobility

Anaesthetic and surgical time >90 minutes

If the surgical procedure is associated with a low risk of major bleeding and taking into account individual risk factors, prophylactic LMWH, or unfractionated heparin for those with severe renal impairment, may be added until mobility is restored. From the risk factors detailed above it can be seen that the majority of head and neck patients are likely to be appropriate for combined pharmacological and mechanical prophylaxis regimes.

**Recommendations**

• Individual assessment for VTE risk and bleeding risk should occur on admission and be reassessed throughout the patient's stay (G)
• Mechanical prophylaxis for VTE is recommended for all patients with one or more risk factors for VTE (R)
• Patients with additional risk factors of VTE and low bleeding risk should have LMWH at prophylactic dose or unfractionated heparin if they have severe renal impairment (R)
